# History of near‐isogenic lines carrying genes for resistance to bacterial blight and blast in rice (*Oryza sativa* L.)

**DOI:** 10.1002/tpg2.70300

**Published:** 2026-09-12

**Authors:** Van Schepler‐Luu, Mary Jeanie Telebanco‐Yanoria, Hiroki Saito, Casiana M. Vera Cruz

**Affiliations:** ^1^ International Rice Research Institute Los Banos The Philippines; ^2^ Japan International Research Center for Agricultural Sciences Ishigaki Japan

## Abstract

Bacterial blight and blast, caused by *Xanthomonas oryzae* pv. *oryzae* and *Magnaporthe oryzae*, respectively, are the two most devastating diseases of rice (*Oryza sativa* L.). Although the diseases were first documented in the 17th–19th centuries, systematic research on their pathology and host resistance began in the early 20th century and was significantly accelerated following the development of near‐isogenic lines (NILs) carrying single resistance genes in universally susceptible backgrounds in the 1980s. Sets of NILs for bacterial blight (IRBB) and International Rice Research Institute (IRRI)‐bred Blast‐resistant Line (IRBL) were developed through a long‐term collaboration between the IRRI and the Japan International Research Center for Agricultural Sciences. Over the years, these materials have been widely distributed for pathogen population monitoring, studying the molecular mechanisms of resistance, assessing the synergistic effect of multiple resistance genes, and developing new resistant varieties, thereby benefiting millions of rice farmers worldwide. In this article, we describe the historical development of the IRBB and IRBL lines, highlight their global impact, and provide perspectives on their relevance not only for bacterial blight and blast but also for emerging rice diseases that could benefit from this approach.

## Core Ideas

- Historical collaboration between International Rice Research Institute and Japan International Research Center for Agricultural Sciences led to the development of near‐isogenic lines (NILs) for bacterial blight (IRBB) and rice blast (IRBL), beginning in the late 1970s and 1980s.
- IRBB and IRBL lines carry single resistance (*R*) genes in a uniform genetic background, enabling standardized evaluation of pathogen populations and resistance.
- These NILs have become global reference materials, widely used for race identification, resistance gene characterization, and breeding in national programs.
- The IRBB and IRBL systems represent a model for international scientific cooperation, resource‐sharing, and capacity building in plant disease resistance.

## Plain Language Summary

Bacterial blight and blast are the two most destructive diseases of rice, threatening global food security. Systematic study on pathogen variation and inheritance of resistance to these diseases accelerated in the early 20th century due to the development of near‐isogenic resistance gene lines (NILs) for bacterial blight (IRBB) and blast (IRBL) in the late 1980s through the collaboration between the International Rice Research Institute (IRRI) and the Japan International Research Center for Agricultural Sciences. The NILs carry single resistance genes in a common susceptible background and serve as global reference materials for pathogen monitoring, molecular studies of host resistance, and breeding for resistant varieties. The widespread use of IRBB and IRBL lines has contributed to improved disease management for millions of farmers worldwide. The success of NILs for bacterial blight and blast provides a model for resistance breeding and surveillance of emerging diseases.

**Abbreviations**
BB: bacterial blight
IRBB: IRRI bacterial blight near‐isogenic line
IRBL: IRRI‐bred blast‐resistant line
IRRI: International Rice Research Institute
JIRCAS: Japan International Research Center for Agricultural Sciences
NIL: near‐isogenic line
TAL: transcription activator‐like effectors
Xoo: *Xanthomonas oryzae* pv. *oryzae*

## 1 INTRODUCTION

Near‐isogenic lines (NILs) carrying a single resistance gene in a common genetic background have revolutionized pathology of rice (*Oryza sativa* L.). By enabling clear, reproducible tests of pathogen virulence and host resistance, these lines are essential for:

- understanding pathogen populations and their virulence mechanisms,
- assessing synergistic interactions of combined resistance genes,
- identifying effective resistance genes for deployment, and
- providing valuable resistance sources for breeding programs worldwide.

**FIGURE 1:**
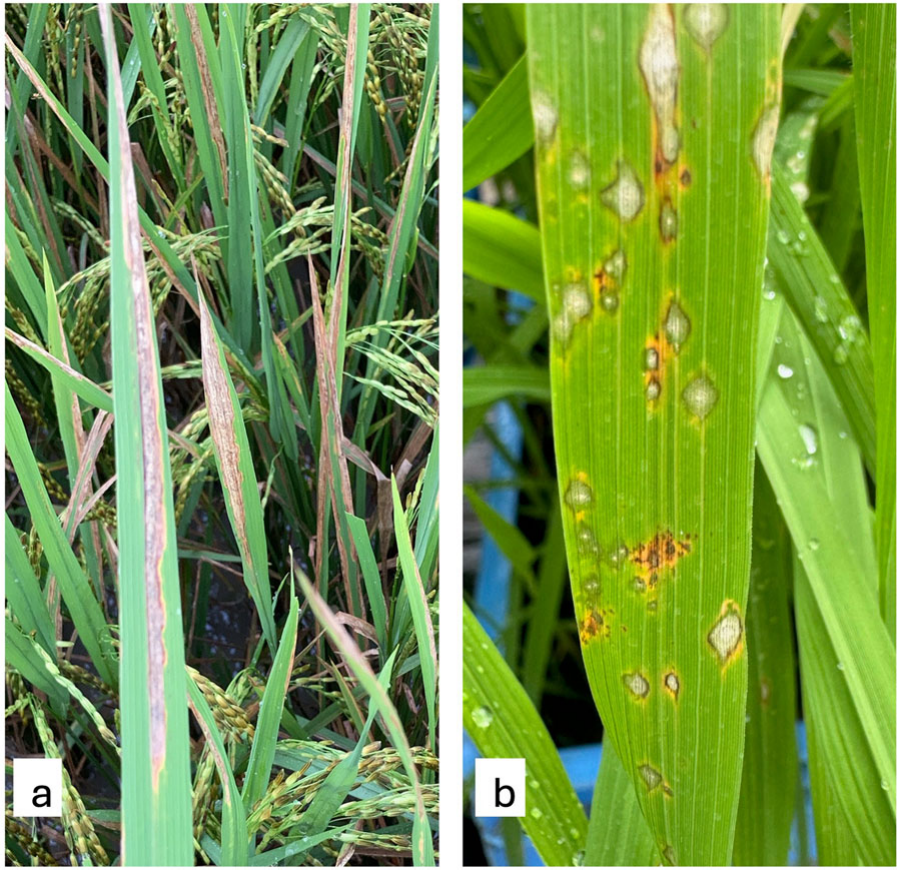
Typical symptoms of bacterial blight and leaf blast. (a) Bacterial blight infection is characterized by wavy margin, yellowing, and wilting of rice leaves. (b) Blast infection showing typical diamond‐shaped lesions on rice leaves.

The IRBB (for bacterial blight) and IRBL (for rice blast, where) NILs, developed through decades of collaboration between the International Rice Research Institute (IRRI) and the Japan International Research Center for Agricultural Sciences (JIRCAS), have become vital tools in nearly every rice pathology laboratory worldwide. Between 2012 and 2025 alone, the International Rice Genebank distributed over 1149 samples of these NILs to 26 institutions across 14 countries, underscoring their significant global impact. The distribution of these NILs to institutions was intended for use in their basic foundational research programs to understand the population biology of *Xanthomonas oryzae* pv. *oryzae* (*Xoo*) and *Magnaporthe oryzae* (*Mo*), the causal agents of bacterial blight (BB) and blast, respectively (Figure 1). The NILs were also used for surveillance and monitoring activities to detect pathogen population shifts and adaptation in released varieties, to study the specific and non‐specific interaction in the rice‐*Xoo* and rice‐*Mo* pathosystems, and to clarify mechanisms associated with transcription activator‐like effectors (TALs) in BB and nucleotide‐binding leucine‐rich repeat proteins in blast resistance. Breeders typically utilize these NILs as genetic resources to enhance the resistance of breeding lines intended for the commercial release of varieties that lack effective resistance (*R*) genes against BB or blast. The resulting improved varieties provide economic benefits to farmers by enabling the cultivation of high‐yielding rice without the need for hazardous chemical applications, particularly for blast disease management.

## 2 ORIGINS AND DEVELOPMENT OF THE IRBB

The concept of BB NILs emerged in the late 1970s during the first visit of IRRI Director General Dr. Nyle C. Brady to Japan's JIRCAS (formerly Tropical Agriculture Research Center). Invited by the Japanese Ministry of Agriculture, Forestry, and Fisheries, Dr. Brady encouraged IRRI scientists to propose collaborative research topics with Japanese counterparts. Among the selected proposals was one from Dr. Tom W. Mew of IRRI's Plant Pathology Department.

Dr. Mew proposed to develop BB NILs carrying resistance (*R*) genes identified at IRRI. The foundation of this research idea is based on early studies on the pathogenic variability or physiologic specialization of *Xoo*. These efforts, which began in the mid‐1970s, confirmed the strong variety‐isolate interaction following H.H. Flor's gene‐for‐gene concept (a pathology concept proposed by Harold Henry Flor that plants and their diseases each have single genes that interact with each other during an infection) in the rice‐*Xoo* pathosystem (Ardales et al., 1996; Leach et al., 1992; Mew, 1987; Mew & Vera Cruz, 1979; Mew et al., 1982, 1992; Ogawa, 1993; Nelson et al., 1994; Vera Cruz et al., 1984; Vera Cruz et al., 2000). *Xoo* is a highly specialized pathogen based on host range, while the host genotypes are race‐ or strain‐specific (Mew, 1987). During the mid‐1970s–1980s, studies on physiological specialization and race specificity in *Xoo* were developed in parallel with systematic evaluation of rice germplasm and differential varieties (DVs). These studies examined variation in resistance and susceptibility, as well as the stability and consistency of lesion expression across plant developmental stages. Resistance expression was influenced by leaf age, plant age, inoculum concentration, and environmental conditions, indicating that host‐pathogen interactions had to be evaluated under standardized conditions. This work enabled the identification and characterization of DVs based on distinct resistance responses, including seedling resistance, moderate resistance, moderate susceptibility, high susceptibility, and adult‐plant resistance, and provided the foundation for later gene‐based differential systems used in *Xoo* pathotyping (Mew et al., 1981; Qi & Mew, 1985). Between 1979 and the mid‐1980s, in the Philippines, six standard *Xoo* strains, PXO61, PXO86, PXO79, PXO71, PXO112, and PXO99, were classified by IRRI to represent the corresponding *Xoo* races 1–6, while seven *Xoo* races, namely IA, IB, II, IIIA, IIIB, IV, and V, were designated in Japan. These strains showed specific interactions on the first set of DVs in the Philippines and Japan: IR20, IR1545‐339‐2‐2, Cas 209, DV85, Tetep, Kogyoku, and Chugoku 45, with IR8, the miracle rice variety, as the susceptible control (Mew & Vera Cruz, 1979; Mew et al., 1982, 1987, 1992; Ogawa & Yamamoto, 1987; Vera Cruz & Mew, 1989). These DVs were later found to contain BB resistance genes: Kogyoku contains *Xa1*, Tetep contains *Xa2*, Chugoku 45/Java14/Zenith/Sateng contains *Xa3*, IR20/IR22 contains *Xa4*; IR1545‐339‐2‐2 contains *xa5*; DV85 contains *xa5* + *Xa7*; and Cas 209 contains *Xa10* (Table 1).

**Core Ideas**

- Historical collaboration between International Rice Research Institute and Japan International Research Center for Agricultural Sciences led to the development of near‐isogenic lines (NILs) for bacterial blight (IRBB) and rice blast (IRBL), beginning in the late 1970s and 1980s.
- IRBB and IRBL lines carry single resistance (*R*) genes in a uniform genetic background, enabling standardized evaluation of pathogen populations and resistance.
- These NILs have become global reference materials, widely used for race identification, resistance gene characterization, and breeding in national programs.
- The IRBB and IRBL systems represent a model for international scientific cooperation, resource‐sharing, and capacity building in plant disease resistance.

**TABLE 1:** Sources of resistance to bacterial blight (BB) used in developing BB near‐isogenic lines (NILs) and pyramids.

Line	*R*‐genes	Chromosome	Donor	IRGC (International Rice Genebank Collection) accession	References
IRBB1	*Xa1*	4	Kogyoku	IRGC 66978	(Sakaguchi, 1967)
IRBB2	*Xa2*	4	Tetep	Unknown	(Sakaguchi 1967)
IRBB3	*Xa3*	11	Chugoku 45	IRGC 525	(Ogawa et al., 1986)
IRBB4	*Xa4*	11	IR20	IRGC 237	(Petpisit et al., 1977)
IRBB5	*xa5*	5	IR1545‐284	IRGC 32624	(Petpisit et al., 1977)
IRBB7	*Xa7*	6	DV85	IRGC 8839	(Sidhu et al., 1978)
IRBB8	*xa8*	7	PI231129	IRGC 11114	(Sidhu et al., 1978)
IRBB10	*Xa10*	11	CAS209	IRGC 15793	(Mew et al., 1982)
IRBB11	*Xa11*	3	IR8	Unknown	(Ogawa & Yamamoto, 1986)
IRBB13	*xa13*	8	BJ1	IRGC 3711	(Ogawa et al., 1987)
IRBB14	*Xa14*	4	TN1	IRGC 105	(Taura et al., 1987)
IRBB21	*Xa21*	11	*O. longistaminata*	Unknown	(Khush et al., 1990)
IRBB23	*Xa23*	11	*O. rufipogon*	Unknown	(Zhang et al., 1998)
IRBB27	*Xa27*	6	*O. minuta*	IRGC 101141	(Gu et al., 2004)

Based on this foundational information, Dr. Mew first proposed to develop BB NIL by introgression of R genes contained in the DVs into a common susceptible background (IR8). These NILs, each carrying individual dominant *Xa* or recessive *xa* genes, can be used as the international differential set for identifying *Xoo* races and guiding breeding programs. He first proposed to use IR8 as the recurrent parent for developing BB NILs. But IR8 was later found to carry the *Xa11* resistance gene (Ogawa & Yamamoto, 1986), prompting the selection of IR24 as the common genetic background for the IRBB NILs. Although later study on inheritance of resistance of IR24 also revealed the presence of *Xa18*, which functions against strain BM8417 and BM8429 in Myanmar but not against PXO86 strain in the Philippines (Yamamoto & Ogawa, 1990).

With support from Japan's Ministry of Foreign Affairs and Ministry of Agriculture, Forestry, and Fisheries, a 5‐year collaborative project was launched in 1985 between IRRI and JIRCAS. This brought together pathologists, breeders, and geneticists from both institutions. Dr. Tsugufumi Ogawa, a geneticist, led NIL development at IRRI's Plant Breeding Department, working closely with IRRI pathologists. Plant pathologist Dr. Tsuyoshi Yamamoto also joined the project, which was co‐led at IRRI by Dr. Mew and Dr. Gurdev S. Khush. Dr. Mew also served as IRRI's project leader for the Japan collaboration for over a decade, until a few years before his retirement in 2004. Visiting scientists Dr. Osamu Horino and Dr. Hisatoshi Kaku contributed to the work, which led to the release of the first IRBB lines published in 1988 and 1991 (Ogawa et al., 1988, 1991). This first set of IRBB lines contains *Xa*/*xa* genes from Kogyoku (*Xa1*), Tetep (*Xa2*), Chugoku 45 (*Xa3*), IR20 (*Xa4*), IR1545‐284 (*xa5*), DV85 (*Xa7*), PI231129 (*xa8*), Cas 209 (*Xa10*), and IR8 (*Xa11*) in IR24 background (Table 1).

Since 1975, IRRI had embarked on an institute‐wide program, “Germplasm Evaluation and Utilization (GEU) Program,” where thousands of germplasm accessions from the International Rice Genebank were screened against biotic and abiotic stresses. This program led to identification of additional varieties and wild rice accessions carrying specific and non‐specific resistance genes to *Xoo* races, including *xa13* (from BJ1) (Ogawa et al., 1987), *Xa14* (from TN1) (Taura et al., 1987), *Xa21* (from *Oryza longistaminata*) (Khush et al., 1990), *Xa23* (from *Oryza rufipogon*) (Zhang et al., 1998), and *Xa27* (from *Oryza minuta*) (Gu et al., 2004) (Table 1). These genes were introgressed into IR24 to create additional IRBB lines (Table 1). Newly identified *Xa* genes from wild rice, including *Xa48*, *xa45(t)*, *Xa38*, and *Xa33*, provided durable resistance against evolving *Xoo* races and could be introgressed into IR24 and other suitable genetic backgrounds (Lore et al., 2025).

Interesting fact: at the time when IRBB NILs were developed, none of the *Xa/xa* genes were cloned, and there was much less information related to the genes and gene markers that could be used for identification of the introgression lines. The introgression was done based on phenotypic screening using the H.H. Flor's gene‐for‐gene principle. The IRBB NILs can be distinguished from each other using the 10 *Xoo* races from the Philippines (Table S1) (Cottyn & Mew, 2004; Vera Cruz et al., 2000; Zhang et al., 2001). And the identification of differential *Xoo* isolates to distinguish different *Xa* genes was crucial to screen and select for the introgression line. On the other hand, different *Xoo* races can also be identified using IRBB NILs carrying specific *Xa* or *xa* genes for BB resistance. Hence, these NILs have since served as the international differential set for identifying *Xoo* races in Asia, Africa, and beyond (Ogawa et al., 1991). To the present day, surveillance and monitoring of pathogen population in BB‐infected fields or fields grown to IR24 and IRBB NILs are continued across rice‐growing countries globally to document *Xoo* evolution over time (Figure S1).

 Later studies on BB demonstrated adaptation of *Xoo* to single gene‐deployed *R* gene *Xa4* (Ardales et al., 1996; Mew et al., 1992; Quibod et al., 2020). Thus, the need for combining effective genes for deployment against predominant pathogen populations has been recommended and employed in the breeding program (Dossa et al., 2015; Lore et al., 2025). When *Xa* gene markers (STS and SNPs) were developed, pyramiding of *Xa* genes progressed rapidly (Huang et al., 1997). More refined *Xa* gene markers were also developed and subsequently applied in breeding programs across Asia (Leung et al., 2004). The *indica* variety IR24 served as the genetic background for all single and pyramided *Xa* genes in various combinations since it has a good combining ability when used as parent in crossing (Leung et al., 2004). Other than IR24, pyramided effective *Xa*/*xa* genes were also developed in IR64 by partners in Asia (Leung et al., 2004). IRRI breeders developed high yielding and BB‐resistant NSIC Rc222 (IRRI 154) carrying *Xa4*+*xa5*, which has been widely grown by farmers in the Philippines due to its disease and pest resistance and high yield. Recently, Platten et al. (unpublished) introgressed several other *Xa* genes into IRRI 154 genetic background, making it suitable for modern breeding. The *Xa* genes and gene combinations available in IRRI 154 background include: *Xa23*, *xa13*, (*Xa21* + *Xa23)*, and (*Xa21* + *Xa23* + *xa25‐2* + *xa41‐Kh*). In some Asian countries like Indonesia, Vietnam, India, Bangladesh, and Pakistan, improved varieties containing effective genes against contemporary pathogen population have been developed and released (Babar et al., 2022; Leung et al., 2004; Lore et al., 2025). In variety Komboka, carrying *Xa4* and (*xa13 + xa25* + *xa41*) edited genes were generated for BB resistance in East and Southern Africa (Schepler‐Luu et al., 2023). Until now, pyramiding effective *Xa* genes remains the most reliable strategy for improving BB resistance in modern rice varieties, but identifying the most suitable genes requires continuous monitoring of dominant pathogen populations.

## 3 ORIGINS AND DEVELOPMENT OF NILS FOR RICE BLAST (IRBL)

When the IRBB project was launched, Dr. Mike Bonman, an IRRI plant pathologist specializing in rice blast, initiated the development of blast NILs using CO39 as the genetic background, in collaboration with Dr. David Mackill, an IRRI breeder and a former head of IRRI's Plant Breeding, Genetics, and Biotechnology Division. The initial set of blast NILs in the susceptible variety, CO39 was published in 1992 (Mackill & Bonman, 1992). From the mid‐1990s to 2010, the IRRI–Japan collaborative project substantially advanced the development of NILs for rice blast under the leadership of Tokio Imbe, Hiroshi Kato, Yoshimichi Fukuta, and Nobuya Kobayashi. By systematically introgressing diverse *Pi* genes, originally identified from different cultivars, into universal susceptible recurrent parents (Tables 2 and 3) (Atkins et al., 1967; Imbe & Matsumoto, 1985; Imbe et al., 1997; Kiyosawa, 1978, 1981, 1984; Mackill & Bonman, 1992; Telebanco‐Yanoria, Koide, Fukuta, Imbe, Kato, et al., 2010; Telebanco‐Yanoria, Koide, Fukuta, Imbe, Tsunematsu, et al., 2010; Tsunematsu et al., 2000; Yamada et al., 1976). A major milestone was the development and characterization of monogenic lines harboring 24 *Pi* genes, designated as IRBLs, in the Lijiangxintuanheigu (LTH) background (Tsunematsu et al., 2000). The IRBLs have uniform genetic backgrounds that are more appropriate for evaluating resistance gene identification, pathotype surveillance, and strategic resistance gene deployment in breeding programs across agroecological regions.

**TABLE 2:** Sources of resistance to blast used in developing the monogenic lines and near‐isogenic lines (NILs).

Line	*R*‐genes	Chromosome	Donor	IRGC accession	References
IRBLsh‐B	*Pish*	1	BL 1	IRGC 40267	(Imbe & Matsumoto, 1985)
IRBLt‐K59	*Pit*	1	K59	IRGC 40261	(Kiyosawa et al., 1981)
IRBLb‐B	*Pib*	2	BL 1	IRGC 40267	(Kiyosawa et al., 1981)
IRBLzt‐T	*Piz‐t*	6	Toride 1	IRGC 40260	(Kiyosawa et al., 1981)
IRBLz5‐CA	*Piz‐5/Pi2*	6	C101A51	IRGC 125596	(Mackill & Bonman, 1992)
IRBLz‐Fu	*Piz*	6	Fukunishiki	IRGC 40257	(Yamada et al., 1976)
IRBL9‐W	*Pi9(t)*	6	WHD‐1S‐75‐1‐127	IRGC 122722	(Amante‐Bordeos et al., 1992)
IRBL3‐CP4	*Pi3*	9	C104PKT	IRTP 16334	(Mackill & Bonman, 1992)
IRBLi‐F5	*Pii*	9	Fujisaka 5	IRGC 2574	(Kiyosawa et al., 1981)
IRBL5‐M	*Pi5(t)*	9	Moroberekan (RIL249)	IRGC 129838	(Wang et al., 1994)
IRBLk‐Ka	*Pik*	11	Kanto 51	IRGC 32562	(Yamada et al., 1976)
IRBL1‐CL	*Pi1*	11	C101LAC	IRTP 16315	(Mackill & Bonman, 1992)
IRBLkh‐K3	*Pik‐h*	11	K3	IRGC 72518	(Kiyosawa, 1978)
IRBLkm‐Ts	*Pik‐m*	11	Tsuyuake	IRGC 40262	(Kiyosawa, 1978)
IRBLkp‐K60	*Pik‐p*	11	K60	Unknown	(Kiyosawa et al., 1981)
IRBL7‐M	*Pi7(t)*	11	Moroberekan (RIL29)	IRGC 129838	(Wang et al., 1994)
IRBLta2‐Pi	*Pita‐2*	12	Pi No4	Unknown	(Kiyosawa, 1967)
IRBLks‐F5	*Pik‐s*	12	Fujisaka 5	IRGC 2574	(Kiyosawa et al., 1981)
IRBLta‐K1	*Pita*	12	K1	unknown	(Kiyosawa et al., 1981)
IRBL12‐M	*Pi12(t)*	12	Moroberekan (RIL29)	IRGC 129838	(Wang et al., 1994)
IRBL20‐IR24	*Pi20(t)*	12	IR24	IRGC 141087	(Imbe et al., 1997)
IRBL11‐Zh	*Pi11(t)*	12	Zhaiyeqing 8	Unknown	(Zhu et al., 1993)
IRBL19‐A	*Pi19(t)*	12	Aichi Asahi	IRGC 40252‐1	(Hayashi et al., 1998)
IRBLa‐A	*Pia*	12	Aichi Asahi	IRGC 40252‐1	(Kiyosawa, 1978, 1981)

**TABLE 3:** International rice blast monogenic and near‐isogenic lines

Type	No. of genes	Resistance genes	Background	References
NIL	4	*Pi1, Pi3, Piz‐5, Pita*	CO39	(Mackill & Bonman, 1992)
ML^a^	24	*Pish, Pib, Pit, Pia, Pii, Pi3, Pi5(t), Pik‐s, Pik‐m, Pi1, Pik‐h, Pik, Pik‐p, Pi7(t), Pi9, Piz, Piz‐5/Pi2(t), Piz‐t, Pita‐2, Pi12(t), Pita, Pi19(t), Pi20(t), Pi11(t)*	LTH	(Tsunematsu et al., 2000)
NIL	11	*Pib, Piz‐5, Pi9, Pi3, Pia, Pik‐s, Pik, Pik‐h, Pi7(t), Pita, Pita‐2*	LTH	(Telebanco‐Yanoria, Koide, Fukuta, Imbe, Kato, et al., 2010)
NIL	14	*Pish, Pib, Piz‐5, Piz‐t, Pi5(t), Pik‐s, Pik, Pik‐h, Pik‐m, Pik‐p, Pi1, Pi7(t), Pita, Pita‐2*	CO39	(Telebanco‐Yanoria, Koide, Fukuta, Imbe, Tsunematsu, et al., 2010)
NIL	18	*Pia, Pii, Pi3, Pi5(t), Pik‐s, Pik‐m, Pi1, Pik‐h, Pik, Pik‐p, Pi7(t), Pi9, Piz, Piz‐5/ Pi2(t), Piz‐t, Pita‐2, Pita/Pi4(ta), Pi20(t)*	US‐2	(Fukuta, Koide et al., 2022)
NIL	6	*Pi35, pi21, Pi37(t), Pb1, Pi34*, *PiPHL9*,	US‐2	(Saito et al., 2022)
NIL	9	*Pi9, Piz‐5, Pi3, Pi5, Pi7, Pik, Pish, Pita‐2, Pish*	IR49830‐7‐1‐2‐2	(Koide et al., 2011)
NIL	11	*Pik, Pik‐h, Pik‐p, Pi1, Pi7(t), Piz, Piz‐5, Pi9, Pish, Pii, Pi3*	IR64	(Fukuta, Telebanco‐Yanoria et al., 2022)

^a^ML refers to monogenic lines, harboring a single‐gene response to the resistance phenotype, while other traits may differ (Fukuta et al., 2004; Tsunematsu et al., 2000). Near‐isogenic lines (NILs) are lines in which only the specific resistance locus has been introgressed in a recurrent parent through backcrossing. They are genetically almost, but not completely, identical to the recurrent parent because small donor‐derived genomic regions may remain around the target locus or elsewhere in the genome (Telebanco‐Yanoria, Koide, Fukuta, Imbe, Kato, et al., 2010).

The gene‐for‐gene theory has long served as the conceptual framework for understanding host‐pathogen interactions in rice blast, similarly for BB (Ou, 1985; Valent, 2025; Valent et al., 1991). Pathotype classification based on differential host reactions remains a standard approach for analyzing blast population structure (Bonman et al., 1986; Ling & Ou, 1969). However, unlike in the case of BB, there was no available set of standard differential blast isolates in the Philippines for resistance studies. Prior to the development of IRBL, it was necessary to select specific *Mo* isolates corresponding to each targeted resistance gene to properly apply the gene‐for‐gene concept within the rice‐blast pathosystem. A total of 119 isolates exhibiting unique pathogenicity reactions against the first‐developed CO39 NILs by Mackill and Bonman in 1992 were selected from earlier studies conducted at the IRRI Plant Pathology. The use of CO39 NILs has been instrumental in dissecting race‐specific resistance and characterizing avirulence–resistance gene interactions in *Mo* (Mackill & Bonman, 1992; Telebanco‐Yanoria, Koide, Fukuta, Imbe, Kato, et al., 2010). The isolates were initially classified based on their reaction patterns to a set of DVs carrying 18 known resistance genes (Table S2). At first, the isolates were grouped into 31 pathotypes based on their compatibility with nine DVs: Aichi Asahi, Yashiromochi, Shin 2, K59, C104PKT, Fujisaka 5, Toride 1, BL1, and C101A51. These primary groups were further subdivided into 70 pathotypes using the reaction patterns of 10 additional DVs: Kanto 51, K3, Tsuyuake, K60, C101LAC, Reiho, Pi No. 4, Fukunishiki, Caloro, and IR24. From these, 20 isolates showing stable reactions and good sporulation were selected as representative isolates capable of differentiating the DVs (Telebanco‐Yanoria et al., 2008). However, some inconsistencies in reactions on the DVs were observed because the DVs did not share the same genetic background, and some lines may harbor additional resistance genes, which can complicate phenotypic interpretation (Fjellstrom et al., 2004). To further validate the differentiating capacity of the 20 selected isolates, monogenic lines developed in the LTH genetic background carrying 24 resistance genes were used to confirm the reaction patterns (Table S3). Monogenic lines in a uniform genetic background such as LTH provide a more reliable system for confirming gene‐specific resistance reactions and minimizing background genetic effects (Tsunematsu et al., 2000).

As *Magnaporthe* populations continue to evolve, and with climate change further complicating disease dynamics, these shared resources remain relevant today as when they were first developed. However, the genetic backgrounds of blast NILs limit their use in breeding programs due to their physiological and morpho‐agronomic traits that hinder the breeding efficiency; thus, in addition to blast NILs in LTH and CO39 backgrounds, more *indica* background NILs in US2, IR49830‐7‐1‐2‐2, and IR64 were also developed (Table 3; Table S4) (Fukuta, Koide, et al., 2022; Fukuta, Telebanco‐Yanoria, et al., 2022; Koide et al., 2011; Saito et al., 2022).

## 4 DEPLOYMENT OF BB AND BLAST RESISTANCE GENES THROUGH REGIONAL AND INTERNATIONAL NETWORKS

The Asian Rice Biotechnology Network (ARBN) was established in three phases from 1993 to 2004, led by Dr. Rebecca Nelson, Dr. Hei Leung, and Dr. John Bennett. This collaborative network among IRRI and 10 institutions in six Asian countries proved essential for resource sharing and continuous training, enabling breeders and partners, including plant pathologists, entomologists, geneticists, and molecular biologists, to adopt advanced tools from host‐pathogen interaction research and genetic knowledge of local pathogen population and varieties for application in breeding programs. Numerous studies have demonstrated the efficient identification and effective use of resistance genes to enhance resistance to blast and BB (Carrillo et al., 2021; Ramalingam et al., 2003; Zaka et al., 2018). Through ARBN, marker‐assisted analyses of pathogens and host plant resistance have been integrated into national breeding programs, leading to the development of elite and commercial lines carrying multiple disease resistance genes (Leung et al., 2004).

To enhance resistance to diverse *Xoo* races globally, various combinations of *Xa/xa* genes conferring different mechanisms of resistance have been pyramided into the IR24 background (Huang et al., 1997). Furthermore, pyramid lines IRBB61 to IRBB66, incorporating various combinations of *Xa4*, *xa5*, *Xa7*, *xa13*, and *Xa21*, were developed in the year 2000 during the final phase of the ARBN. The pyramid lines with effective gene combinations were shared with Asian partners for improving BB resistance in their popular commercial varieties (Leung et al., 2004). Pyramid lines were released in their respective countries; for example, Angke (carrying *Xa4* + *xa5*) and Conde (with *Xa4* + *Xa7*) were released in 2002 in Indonesia by Masdiar Bustaman from Indonesian Center for Agricultural Biotechnology and Genetic Resources Research and Development. Later studies in Indonesia confirmed the presence of *Xa7* in variety Conde, which continued to exhibit resistance to most of the Indonesian *Xoo* races and high yield (Fatimah et al., 2018). The effectiveness of each gene combination varies depending on the virulence spectrum of *Xoo* races in different rice‐growing regions (Table S1).

Before the early 2000s, rice blast research was conducted mainly at national or regional levels using locally developed DVs and race classification systems. This lack of standardization limited the comparability of resistance evaluations and pathogen virulence data among countries, thereby constraining global understanding of host‐pathogen interactions and impeding coordinated breeding efforts for durable blast resistance. To address these limitations, the Blast Research Network for Stable Rice Production was established in 2006 under the leadership of Dr. Yoshimichi Fukuta, with institutional support from JIRCAS Collaborative Research Project commissioned by Japan's Ministry of Agriculture, Forestry, and Fisheries. The program evolved from earlier Japan‐IRRI collaborative research initiatives and was conceived as an international platform integrating rice breeders, plant pathologists, and geneticists from different countries. Participating countries included major rice‐producing nations in Asia such as Japan, the Philippines, China (Yunnan Agricultural University), Korea, Vietnam, Thailand, Bangladesh, Indonesia, Lao LDR and Cambodia, Kenya, and two institutes (IRRI and AfricaRice). The primary goal of the Blast Research Network is to strengthen the blast research collaboration by (1) evaluating blast resistance using a set of monogenic lines in the LTH background using a standard screening protocol; (2) characterizing *Mo* isolates across regions; and (3) enhancing cooperation among participating countries on their breeding programs.

In parallel with host resistance standardization, Blast Research Network emphasized the coordinated collection and characterization of *Mo* isolates from diverse agroecological regions. Pathogen isolates contributed by participating countries were evaluated using the standardized IRBL set, generating comparable virulence profiles, and facilitating analyses of pathogen population structure, race diversity, and geographic differentiation. These efforts enhanced understanding of blast pathogen evolution and supported informed strategies for resistance gene deployment, including gene pyramiding and region‐specific resistance management.

Institutionally, the “Blast Research Network for Stable Rice Production” activities were closely aligned with JIRCAS projects. Through these mechanisms, IRBL lines, standardized protocols, and technical knowledge were disseminated widely. The network also contributed to capacity building by promoting training, data sharing, and sustained collaboration among national agricultural research systems of the participating institutions. Additionally, each institute developed a differential system consisting of DVs and standard blast isolates, representing a key achievement in pathological research. This system serves as a fundamental tool for characterizing resistance genes and assessing isolate pathogenicity, supporting the genetic improvement of leading rice cultivars.

**FIGURE 2:**
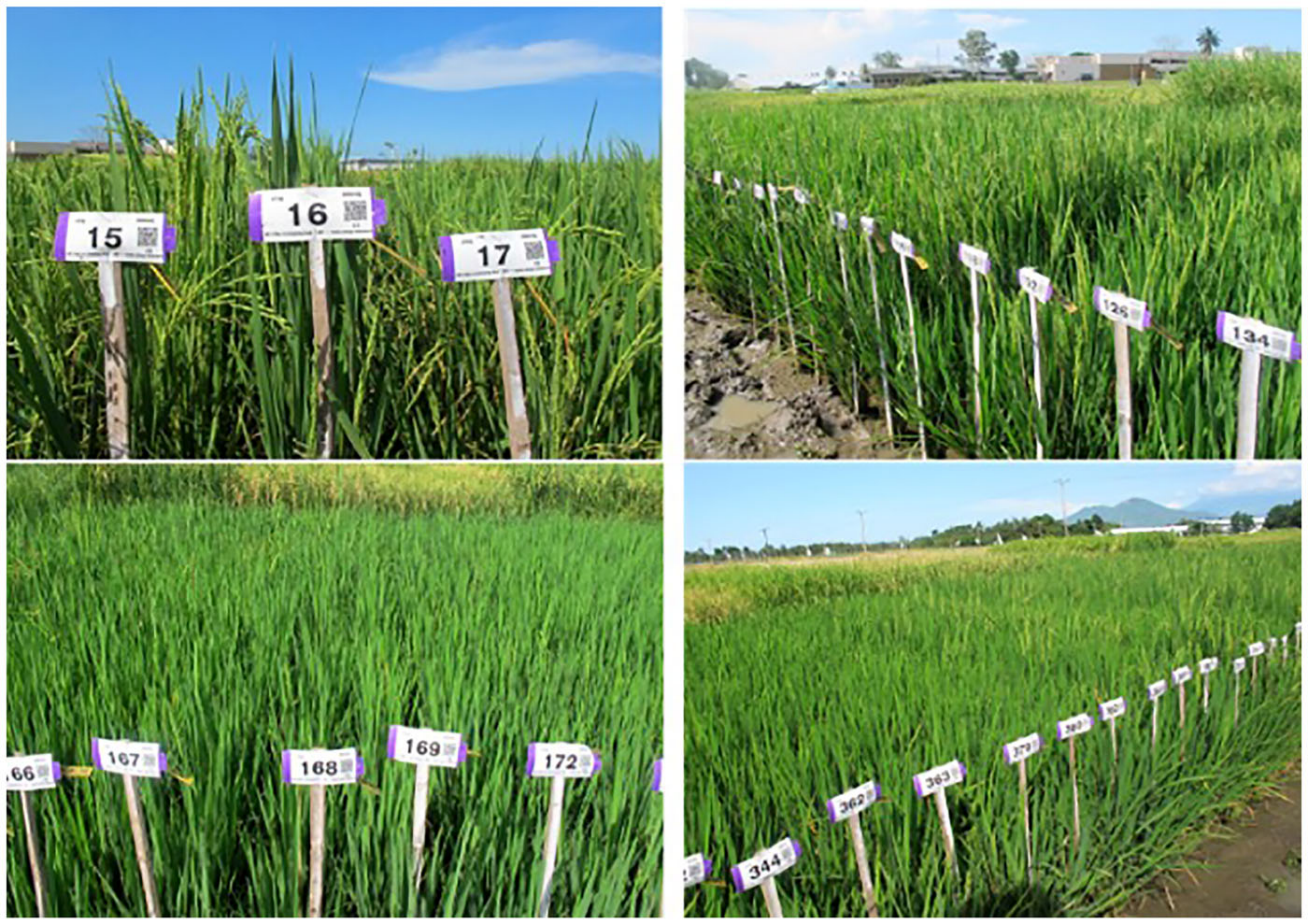
International Rice Research Institute (IRRI) field evaluation of Ciherang Sub1 + AG1 (CSA) lines introgressed with Xa/xa and Pi genes during the 2017 dry season. The CSA breeding lines, with abiotic stress tolerance and resistance to bacterial blight and rice blast, were evaluated for agronomic performance at different crop growth stages.

In 2014, the Stress Tolerant Rice for Africa and South Asia (STRASA) Project Phase III focused on increasing disease resistance of abiotic stress tolerant varieties by pyramiding various combinations of effective *R* genes, like *Xa4*, *xa5*, *xa13*, *Xa21*, *Xa23* in IRBB pyramids and *Pi‐*genes in IRBLs, including *Pita* and *Pi54*, into improved genetic backgrounds. This aimed at combining effective *Xa/xa* and *Pi* genes with abiotic stress tolerance and good grain quality traits. Recipient breeder‐nominated varieties included Ciherang‐Sub1 + AG1 (CSA, with submergence and anaerobic germination tolerance) (Figure 2), Makassane (with good grain quality), and BINA Dhan 10 (with salinity tolerance). The intermediate and elite lines were shared with IRRI breeders and national program partners for advancement, performance evaluation, and selection into elite lines at the collaborative partners’ target deployment regions in Asia and Africa. Simultaneously, to understand the population structure of the contemporary *Xoo* and *Mo* pathogen populations, their dynamics and variation were monitored over time at their target deployment areas using Geographic Information System.

**FIGURE 3:**
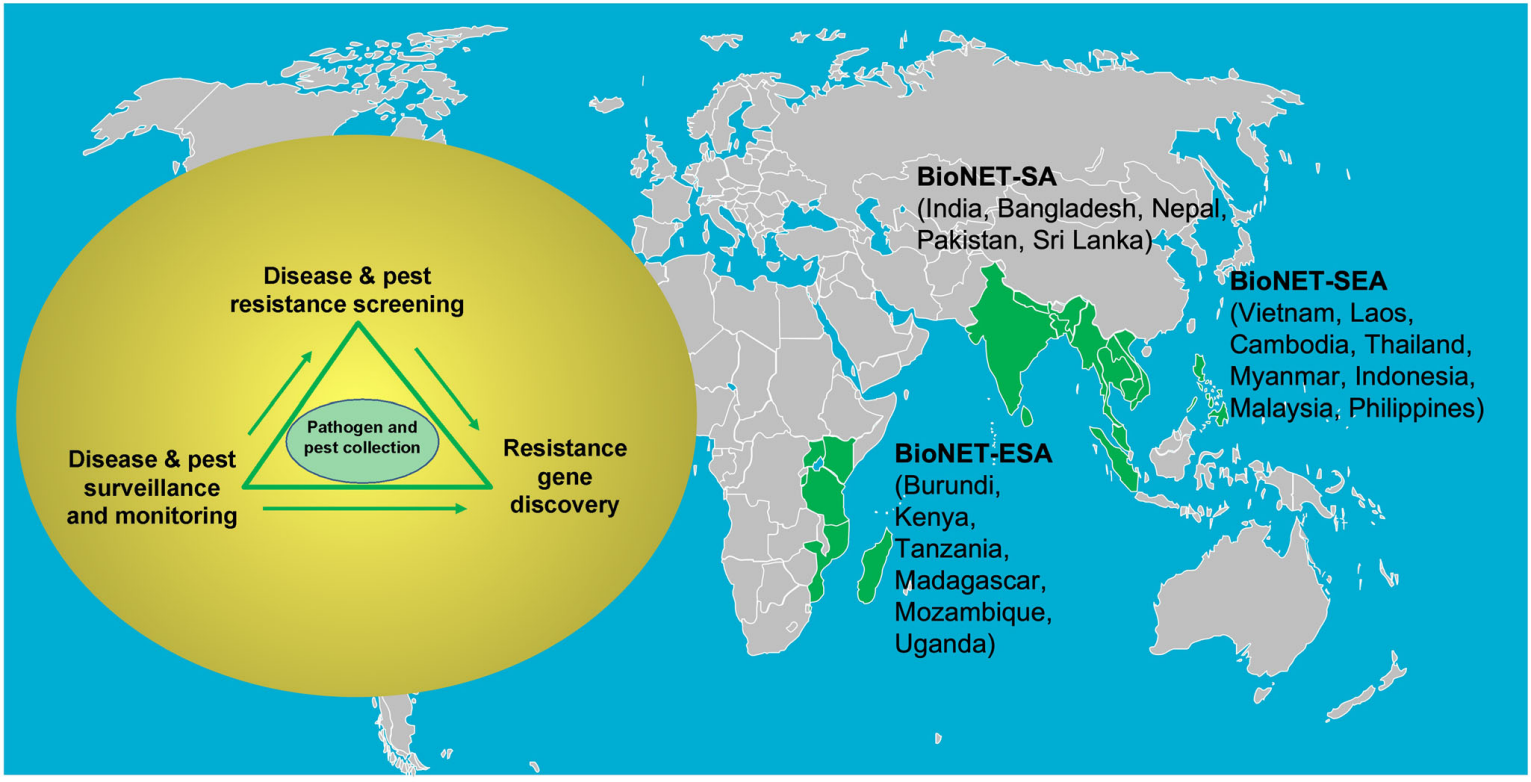
Global Biotic Stresses Screening and Monitoring Network (Global BioNET) established by International Rice Research Institute (IRRI) in 2023 with partners from 16 countries in Southeast Asia (SEA), South Asia (SA), and East and Southern Africa (ESA). The IRRI bacterial blight near‐isogenic line (IRBB) and IRRI‐bred blast‐resistant line (IRBL) have been distributed to global BioNET partners for bacterial blight (BB) and blast resistance monitoring.

More recently, in 2023, the global BioNET (Figure 3), which is a network of rice pathologists and entomologists across 16 countries in Southeast Asia, South Asia, and East and Southern Africa, was established by IRRI under the framework of the CGIAR Plant Health Initiative in order to coordinate the rice pathology activities across national partners. The network aims at (1) coordination of rice disease and pest surveillance and monitoring, (2) developing and sharing protocols and germplasm for resistance screening, and (3) discovery of new disease resistance genes. Global BioNET led by IRRI has become a platform for sharing the new standard set of IRBB (Table 1) and IRBL (Table S4; Figure S2) to national partners to standardize the pathogen surveillance system but also to provide national partners with more elite germplasm for resistance breeding.

## 5 THE GLOBAL LEGACY OF IRBB AND IRBL LINES: WHAT PARTNERS SAY

The IRBB and IRBL developed at IRRI have become global public goods, enabling researchers and breeders across continents to better understand pathogen populations, deploy durable resistance, and improve food security in rice‐growing regions. Until now, pathologists and breeders continued to utilize IRBB and IRBL NILs in their research program as genetic resources to enhance BB and blast resistance. Furthermore, with advances in genomics, new opportunities are emerging to link genetic variation with disease resistance phenotypes and field performance. The outlook for achieving sustainable disease control has been promising, as breeding programs have the capacity to access and apply these tools to address local challenges. Below, partners from around the world share how IRBB and IRBL lines have advanced research, breeding, and impact on the ground:

The near‐isogenic IRBB lines have been instrumental in advancing our understanding of single‐gene resistance in rice. These lines not only enabled the cloning of multiple BB resistance genes but also facilitated the identification, cloning, and functional analysis of corresponding effector genes. Their continued use today underscores their enduring value to the research community. The development of these lines reflects the remarkable foresight of their creators. (Dr. Jan Leach, Colorado State University, USA)

IRBB and IRBL lines have been extensively used in India for breeding varieties resistant to BB and blast, as well as for pathotyping and profiling pathogen races across the country. Some of the resistant varieties developed using IRBB lines include Improved Pusa Basmati‐1, Improved Samba Mahsuri, DRR Dhan 58, and DRR Dhan 60. DRR Dhan 62 and Pusa Samba were developed using IRBL lines. These varieties are widely cultivated across the country. The real impact of IRBB and IRBL lines is felt in the fields of smallholder farmers—reducing crop loss and pesticide use. (Dr. Raman Sundaram, ICAR‐Indian Institute of Rice Research (ICAR‐IIRR), India)

At BRRI, we used IRBB60 as the donor and BRRI Dhan29 as the recipient to develop the BB‐resistant variety BRRI dhan101, released in 2021 for the *Boro* season. It is now grown in farmers’ fields. IRBB60 was also crossed with BRRI Dhan28 to develop two advanced lines with strong resistance in both artificial and hotspot screenings. Genotyping confirmed the presence of *Xa4*, *xa13*, and *Xa21*. Additional crosses have produced three advanced resistant lines containing *Xa4* and *Xa21*, which are now used as donors in our breeding program. (Dr. Shireen Quazi, Bangladesh Rice Research Institute (BRRI), Bangladesh)

In Indonesia, the IRBB lines have greatly contributed to the development of high‐yielding rice varieties resistant to BB. The most recent widely cultivated variety, Inpari 32, released in 2013 from a Ciherang × IRBB64 cross, is resistant to BB pathotype III and moderately resistant to pathotypes IV and VIII. (Dr. Nafisah Nafisah, Indonesian Center for Rice Instrument Standard Testing (ICRIST), Indonesia)

In Madagascar, IRBB lines were used to evaluate local strains of *Xanthomonas oryzae* pv. *oryzae*. Crosses between widely grown cultivars and resistant IRBB lines are now underway to develop new varieties with durable resistance. (Dr. Harinjaka Raveloson, FOFIFA, Centre Régional de Recherches, Madagascar)

As a rice pathologist, I use these NILs to track the emergence of new virulent races and guide breeding decisions that protect farmers’ yields. This is science that directly benefits people on the ground. (Dr. Jennifer Niones, Philippine Rice Research Institute (PhilRice), Philippines)

NILs for BB and rice blast developed by IRRI scientists have been instrumental in dissecting pathogen population dynamics and breeding durable resistance. For us, these NILs served as differential panels to identify changing virulence profiles of *Xanthomonas oryzae* pv. *oryzae* and *Mo* populations in India, and accordingly device pathogen‐informed breeding strategies for broad‐spectrum and durable resistance. By tracking shifts in pathogen population that could defeat specific resistance genes, we are able to anticipate resistance erosion in the field and design effective gene pyramided rice hybrids accordingly. Overall, these NIL panels helped directly to inform the breeding of more durable, multigene resistance in elite backgrounds, thereby accelerating marker assisted introgression and pyramiding of effective combinations tailored to evolving pathogen populations across target countries. (Dr. Deo Mishra, Asia Breeding – Plant Health, Bayer CropScience, India)

IRRI's differential lines have allowed us to map the effectiveness of resistance genes against local pathogen populations with greater accuracy. This regional insight is critical for developing durable disease resistance in commercial rice hybrids. (Dr. Raman Babu, Distinguished Laureate & South Asia Seed Product Development Lead, Corteva Agriscience, India)

## 6 FUTURE PERSPECTIVES

As climate change intensifies disease pressure, the evolution of pathogen populations also accelerates; NILs will remain important resources for rice research, resistance breeding, and pathogen monitoring. Changing temperature, rainfall patterns, humidity, and cropping systems may shift pathogen populations and increase the risk of resistance breakdown, especially when resistance genes are deployed without information on local pathogen diversity. For this reason, future use of IRBB and IRBL materials should be linked more closely with continuous pathogen surveillance and pathogen‐informed deployment strategies. The NILs can therefore function not only as differential materials for pathotyping but also as standardized lines for detecting shifts in the pathogen populations, identifying emerging virulence, and supporting region‐specific deployment of resistance genes.

To sustain and expand their impact, future efforts will focus on five key areas:

- Expanding NIL collections to incorporate novel resistance genes identified through advanced genomics, genome‐wide association studies, and exploration of wild rice relatives.
- Pyramiding multiple resistance genes to enhance the durability of resistance and slow down pathogen adaptation, ensuring long‐term effectiveness in farmers' fields.
- Integrating marker‐assisted and genomic selection into breeding pipelines to accelerate the deployment of NIL‐derived resistance traits in elite varieties.
- Developing NILs for additional stresses, such as rice yellow mottle virus, sheath blight, tungro, and major abiotic challenges, to address the broader spectrum of constraints faced by rice farmers.
- Strengthening international networks such as the International Rice Blast Research Network and global BioNET and supporting new pathogen monitoring systems to promote data sharing, harmonize evaluation protocols, and build global capacity in disease surveillance.

The success of the IRBB and IRBL lines stands as a compelling testament to the power of global scientific collaboration. These resources, and the partnerships behind them, have helped secure food systems in the past and will continue to play a critical role in safeguarding rice production amid emerging challenges. Beyond rice, this strategy offers valuable lessons for other crops in harnessing plant–microbe interactions to build durable resistance and secure food production.

## AUTHOR CONTRIBUTIONS

**Van Schepler‐Luu**: Conceptualization; funding acquisition; investigation; methodology; resources; supervision; visualization; writing—original draft; writing—review and editing. **Mary Jeanie Telebanco‐Yanoria**: Data curation; formal analysis; investigation; resources; validation; visualization; writing—original draft; writing—review and editing. **Hiroki Saito**: Conceptualization; data curation; investigation; resources; visualization; writing—review and editing. **Casiana M. Vera Cruz**: Conceptualization; resources; visualization; writing—original draft; writing—review and editing.

## CONFLICT OF INTEREST STATEMENT

The author declare no conflicts of interest.

## Supporting information



Supplementary Material

## Data Availability

All data for this article is provided in Tables and Figures
