# Supplementary material for: History of near‐isogenic lines carrying genes for resistance to bacterial blight and blast in rice (Oryza sativa L.)
Source: Plant Genome. 2026 Sep 12;19(3):e70300. doi: 10.1002/tpg2.70300 (PMC13570268; doi:10.1002/tpg2.70300)
Supplement: Supplementary file 1 [file TPG2-19-e70300-s001.docx]

**History of Near-Isogenic Lines Carrying Genes for Resistance to Bacterial Blight and Blast in Rice** **(*Oryza sativa* L.)**

**Supplementary figures**

**Supplementary Figure 1**: IR24 (susceptible check) and IRBB57 (carrying *Xa* resistance gene(s) at the maturity stage during bacterial blight (BB) monitoring in the Philippines. IRBB near-isogenic lines were deployed in BB hotspot sites to monitor the field population of *Xoo* and evaluate the durability and effectiveness of *Xa* resistance genes under natural infection.

**Supplementary Figure 2**. Seed multiplication of IRBLs for global BioNET in IRRI’s field (2026 Dry season)

**Supplementary Tables**

**Suppl. Table 1. IRBB lines and pyramids developed through IRRI-JIRCAS collaboration showing specific interactions with differential races of the Philippines Xanthomonas oryzae pv. oryzae.**

| **Line** | ***R-genes*** | **Race 1** | **Race 2** | **Race 3B** | **Race 3C** | **Race 4** | **Race 5** | **Race 6** | **Race 7** | **Race 8** | **Race 9a** | **Race 9b** | **Race 9c** | **Race 9d** | **Race 10** |
| --- | --- | --- | --- | --- | --- | --- | --- | --- | --- | --- | --- | --- | --- | --- | --- |
|  |  | **PXO 61b** | **PXO86** | **PXO79** | **PXO340** | **PXO71** | **PXO112** | **PXO99** | **PXO145** | **PXO280** | **PXO339** | **PXO349** | **PXO347** | **PXO363** | **PXO341** |
| IRBB1 | *Xa1* | S | S | S | S | S | S | S | S | S | S | S | S | S | S |
| IRBB3 | *Xa3* | S | S | S | S | S | S | S | S | S | S | S | S | S | S |
| IRBB4 | *Xa4* | R | S | S | S | MS | R | S | R | R | S | S | S | S | R |
| IRBB5 | *xa5* | R | R | R | R | S | R | S | R | R | R | R | R | R | R |
| IRBB7 | *Xa7* | MS | R | R | R | S | R | S | R | R | S | MS | S | MS | MS |
| IRBB8 | *xa8* | S | S | S | S | S | MR | S | S | S | S | S | S | S | S |
| IRBB10 | *Xa10* | S | R | S | S | S | R | S | R | S | S | S | S | S | S |
| IRBB11 | *Xa11* | S | S | S | S | S | S | S | S | S | S | S | S | S | S |
| IRBB13 | *xa13* | S | S | S | S | S | S | R | S | S | S | S | S | S | S |
| IRBB14 | *Xa14* | S | S | S | S | S | R | S | S | R | S | S | S | S | S |
| IRBB21 | *Xa21* | R | R | R | R | R | R | R | R | R | R | R | R | R | S |
| IRBB23 | *Xa23* | R | R | R | R | R | R | R | R | R | R | R | R | R | R |
| IRBB50 | *Xa4 + xa5* | R | R | R | R | R | R | S | R | R | R | R | R | R | R |
| IRBB51 | *Xa4 + xa13* | R | S | S | S | MS | R | R | R | MR | S | - | - | - | R |
| IRBB52 | *Xa4 + Xa21* | R | R | R | MR | MR | R | MR | R | R | MR | - | - | - | R |
| IRBB53 | *xa5 + xa13* | - | - | - | - | - | - | - | - | - | - | - | - | - | - |
| IRBB54 | *xa5 + Xa21* | R | R | R | R | R | R | R | R | R | R | - | - | - | R |
| IRBB55 | *xa13 + Xa21* | R | R | R | MR | R | R | R | MR | MR | MR | - | - | - | S |
| IRBB56 | *Xa4 + xa5 + xa13* | R | R | R | R | R | R | MR | R | R | R | - | - | - | R |
| IRBB57 | *Xa4 + xa5 + Xa21* | R | R | R | R | R | R | R | R | R | R | - | - | - | R |
| IRBB59 | *xa5 + xa13 + Xa21* | R | R | R | R | R | R | R | R | R | R | - | - | - | R |
| IRBB60 | *Xa4 + xa5 + xa13 + Xa21* | R | R | R | R | R | R | R | R | R | R | - | - | - | R |
| IRBB61 | *Xa4 + xa5 + Xa7* | R | R | R | R | MR | R | S | R | R | R | - | - | - | R |
| IRBB62 | *Xa4 + Xa7 + Xa21* | R | R | R | R | R | R | MR | R | R | MS | - | - | - | R |
| IRBB63 | *xa5 + Xa7 + xa13* | R | R | R | R | R | R | R | R | R | R | - | - | - | R |
| IRBB64 | *Xa4 + xa5 + Xa7 + Xa21* | R | R | R | R | R | R | R | R | R | R | - | - | - | R |
| IRBB65 | *Xa4 + Xa7 + xa13 + Xa21* | R | R | R | R | R | R | R | R | R | MR | - | - | - | R |
| IRBB66 | *Xa4 + xa5 + Xa7 + xa13 + Xa21* | R | R | R | R | R | R | R | R | R | R | - | - | - | R |
| IRBB67 | *Xa4 + Xa7* | - | - | - | - | - | - | - | - | - | - | - | - | - | - |
| IR24 | *Xa18 (susceptible recurrent)* | S | S | S | S | S | S | S | S | S | S | S | S | S | S |
| Resistance or susceptibility of rice plants to *Xanthomonas oryzae* pv. *oryzae* is expressed in lesion lengths measured 14 days post inoculation. Resistant (R): <5 cm; moderately resistant (MR): 5 – 10 cm; moderately susceptible (MS): 10 – 15 cm; susceptible (S): >15 cm. All NILs were inoculated at 40-45 days after sowing. This table includes representative strain of each race of Xanthomonas oryzae pv. oryzae in the Philippines. | | | | | | | | | | | | | | | |

| **Suppl.Table 2. Differential varieties used in the pathogenicity test of *M. oryzae* for the selection of differential isolates** | | |
| --- | --- | --- |
| Differential Varieties | Resistance Genes | Reference |
| Zenith, Aichi Asahi | *Pia* | Atkins *et al*., 1967, Yamada *et al*., 1976, Kiyosawa (1978, 1981, 1984) |
| BL1 | *Pib* | Kiyosawa (1978, 1981, 1984) |
| Fujisaka 5 | *Pii* | Yamada *et al*., 1976, Kiyosawa (1978, 1981, 1984) |
| Kanto 51 | *Pik* | Yamada *et al*., 1976 |
| Tsuyuake | *Pik-m* | Yamada *et al*. 1976, Kiyosawa (1978, 1981, 1984 |
| K60 | *Pik-p* | Kiyosawa (1978, 1981, 1984) |
| K3 | *Pik-h* | Kiyosawa (1978, 1981, 1984) |
| Caloro | *Pik-s* | Atkins *et al.,* 1967 |
| Shin2 | *Pish* | Yamada *et al*., 1976, Kiyosawa (1978, 1981, 1984) |
| K59 | *Pit* | Kiyosawa (1978, 1981, 1984) |
| Reiho, Pi No.4 | *Pita-2* | Yamada *et al*. 1976, Kiyosawa (1978, 1981, 1984) |
| Fukunishiki | *Piz* | Yamada *et al*. 1976, Kiyosawa (1978, 1981, 1984) |
| Toride 1 | *Piz-t* | Mackill & Bonman 1992 |
| C101A51 | *Piz-5=Pi2* | Mackill & Bonman 1992 |
| C101LAC | *Pi1* | Mackill & Bonman 1992 |
| C104PKT | *Pi3* | Mackill & Bonman 1992 |
| Yashiromochi | *Pita* | Yamada *et al*., 1976 |
| IR24 | *Pi20(t)* | Imbe *et al*., 1997 |

**Suppl. Table 3.  Differential reactions of monogenic lines (IRBL) against the standard differential M. oryzae isolates from the Philippines (Telebanco-Yanoria et al., 2008)**

| Target gene | Designation^a^ | Blast isolates and pathotypes^b^ | | | | | | | | | | | | | | | | | | | |
| --- | --- | --- | --- | --- | --- | --- | --- | --- | --- | --- | --- | --- | --- | --- | --- | --- | --- | --- | --- | --- | --- |
|  |  | Ca89 | Ca41 | IK81-3 | M64-1-3-9-1 | M39-1-3-8-1 | V850256 | V86010 | BN111 | PO6-6 | M39-1-2-21-2 | JMB8401 | 43 | M36-1-3-10-1 | BN209 | M101-1-2-9-1 | IK81-25 | JMB840610 | V850196 | B90002 | C923-49 |
|  |  | Ib | Id | II | IIIa | IIIa | IIIc | IVe | Va | Vb | VIb | XIIa | XVIII | XIXb | XXIIa | XXIIa | XXIII | XXIVb | XXVII | XXVIIIb | XXVIIIb |
| *Pia* | IRBLa-C | S | S | S | S | S | S | S | S | S | S | S | S | S | S | S | S | S | S | R | R |
| *Pita* | IRBLta-K1 | S | R | R | S | R-M | R | S | S | MS | S | M | S | MS | S | S | R | S | R | R | S |
| *Pik-s* | IRBLks-F5 | S | S | S | S | S | S | R | S | S | S | S | S | S | S | S | S | S | R | S | S |
| *Pit* | IRBLt-K59 | S | S | S | S | S | S | S | S | S | S | S | S | S | S | S | S | S | S | S | S |
| *Pi3* | IRBL3-CP4 | S | S | S | S | M | S | S | R-M | R | R | R | R | S | S | S | R | S | R | S | S |
| *Pii* | IRBLi-F5 | S | S | S | S | M | S | S | R | R | R-M | R | R | S | S | S | R | S | R | S | S |
| *Piz-t* | IRBLzt-T | S | S | S | R | R | S | R | S | S | S | S | S | S | S | S | S | R-M | S | R | R |
| *Pib* | IRBLb-B | S | S | S | S | S | S | M | S | S | S | S | S | S | R-M | R | S | R | S | R | R |
| *Piz-5* | IRBLz5-CA | M | R | R-M | M | M | M | M | R | R | R-M | R | M | R | M | S | R | R | R-M | M | M |
| *Pita-2* | IRBLta2-Re | S | R | R | R-M | R | R | R | R | S | R | R | S | R | R | R | R | R | R | R | S |
| *Piz* | IRBLz-Fu | M | M | R | R-M | R-M | M | R-M | S | R-M | R-M | R-M | R | R | R-M | R | R | M | R | M | R |
| *Pik* | IRBLk-Ka | R | R | R | S | S | R | R | R | R | S | S | R | S | R | R | S | R | R | R | R |
| *Pi1* | IRBL1-CL | R | R | R | S | S | R | R | R | R | S | S | R | S | R | R | S | R | R | R | R |
| *Pik-h* | IRBLkh-K3 | R | R | R | S | S | R | R | R | R | S | S | R | MS | R | R | M | R | R | R | R |
| *Pik-m* | IRBLkm-Ts | R | R | R | S | S | R | R | R | R | S | S | R | M | R | R | MS | R | R | R | R |
| *Pik-p* | IRBLkp-K60 | R | R | R | S | S | R | R | R | R | S | S | R | S | R | R | S | R | R | R | R |
| *Pi20*(t) | IRBL20-IR24 | S | S | S | S | S | S | R | R | S | R | S | S | R | S | S | MS | S | S | R | R |
| *Pi5*(t) | IRBL5-M | S | M | R-M | M | R | MS | S | R-M | R | R | R | R | R-M | MS | R | R | M | R | M | MS |
| *Pi7*(t) | IRBL7-M | R | R | R | S | S | R | S | R | R | S | S | R | S | R | R | S | R | R | R | R |
| *Pi9* | IRBL9-W | R | R | R | R-M | M | R | S | R | R | R-M | R | R | R | R | R | R | R | R-M | R | R |
| *Pi11*(t) | IRBL11-Zh | S | S | M | S | S | S | R | S | S | S | MS | S | MS | R-M | R | S | R | S | R | R |
| *Pi12*(t) | IRBL12-M | S | S | S | S | S | S | M | S | S | S | S | S | S | R | R | S | R | M | R | R |
| *Pi19*(t) | IRBL19-A | S | S | S | S | S | S | S | S | S | S | MS | S | MS | S | S | S | S | S | S | S |
| *Pish* | IRBLsh-S | M | R-M | R-M | M | M | R-M | M | R-M | R-M | R | S | M | R | M | R-M | R | M | R-M | R-M | M |
| a Monogenic lines are designated as IRBL, followed by the resistance genes, and the abbreviation of the resistant donor variety | | | | | | | | | | | | | | | | | | | | | |
| b R: resistant; M: moderate resistant; MS: moderate susceptible; S: susceptible; R-M: varied from resistant to moderate resistant | | | | | | | | | | | | | | | | | | | | | |

**Suppl. Table 4. List of all available blast monogenic/near-isogenic lines (provided by Dr. Nobuya Kobayashi)**

| **Recurrent parent and no. of genes** | **Serial Number** | **Designation in recent publications** | **Seed source (JIRCAS No.)** | **Rice blast resistant genes in recent publications** | **Gene donor accession name** | **References** |
| --- | --- | --- | --- | --- | --- | --- |
| LTH Monogenic Lines (24 genes) | 1 | Lijiangxintuanheigu  (LTH) | BMsf-1 | *no gene* | *-* | Tsunematsu *et al.,* 2000 |
|  | 2 | IRBLsh-B | BMsf-2 | *Pish* | BL1 | Tsunematsu *et al.,* 2000 |
|  | 3 | IRBLsh-S | BMsf-3 | *Pish* | Shin 2 | Tsunematsu *et al.,* 2000 |
|  | 4 | IRBLb-B | BMsf-4 | *Pib* | BL1 | Tsunematsu *et al.,* 2000 |
|  | 5 | IRBLt-K59 | BMsf-5 | *Pit* | K59 | Tsunematsu *et al.,* 2000 |
|  | 6 | IRBLa-A | BMsf-6 | *Pia* | Aichiasahi | Tsunematsu *et al.,* 2000 |
|  | 7 | IRBLa-C | BMsf-7 | *Pia* | CO 39 | Tsunematsu *et al.,* 2000 |
|  | 8 | IRBLi-F5 | BMsf-8 | *Pii* | Fujisaka 5 | Tsunematsu *et al.,* 2000 |
|  | 9 | IRBL3-CP4 | BMsf-9 | *Pi3* | C104PKT | Tsunematsu *et al.,* 2000 |
|  | 10 | IRBL5-M | BMsf-10 | *Pi5(t)* | RIL249 | Tsunematsu *et al.,* 2000 |
|  | 11 | IRBLks-F5 | BMsf-11 | *Pik-s* | Fujisaka 5 | Tsunematsu *et al.,* 2000 |
|  | 12 | IRBLks-S | BMsf-12 | *Pik-s* | Shin 2 | Tsunematsu *et al.,* 2000 |
|  | 13 | IRBLkm-Ts | BMsf-13 | *Pik-m* | Tsuyuake | Tsunematsu *et al.,* 2000 |
|  | 14 | IRBL1-CL | BMsf-14 | *Pi1* | C101LAC | Tsunematsu *et al.,* 2000 |
|  | 15 | IRBLkh-K3 | BMsf-15 | *Pik-h* | K3 | Tsunematsu *et al.,* 2000 |
|  | 16 | IRBLk-Ka | BMsf-16 | *Pik* | Kanto 51 | Tsunematsu *et al.,* 2000 |
|  | 17 | IRBLkp-K60 | BMsf-17 | *Pik-p* | K60 | Tsunematsu *et al.,* 2000 |
|  | 18 | IRBL7-M | BMsf-18 | *Pi7(t)* | RIL29 | Tsunematsu *et al.,* 2000 |
|  | 19 | IRBL9-W | BMsf-19 | *Pi9* | WHD-1S-75-1-127 | Tsunematsu *et al.,* 2000 |
|  | 20 | IRBLz-Fu | BMsf-20 | *Piz* | Fukunisiki | Tsunematsu *et al.,* 2000 |
|  | 21 | IRBLz5-CA-1 | BMsf-21 | *Piz-5/ Pi2*(t) | C101A51 | Tsunematsu *et al.,* 2000 |
|  | 22 | IRBLzt-T | BMsf-22 | *Piz-t* | Totide 1 | Tsunematsu *et al.,* 2000 |
|  | 23 | IRBLta2-Pi | BMsf-23 | *Pita-2* | Pi No.4 | Tsunematsu *et al.,* 2000 |
|  | 24 | IRBLta2-Re | BMsf-24 | *Pita-2* | Reiho | Tsunematsu *et al.,* 2000 |
|  | 25 | IRBL12-M | BMsf-25 | *Pi12(t)* | RIL10 | Tsunematsu *et al.,* 2000 |
|  | 26 | IRBLta-CP1 | BMsf-26 | *Pita* | C101PKT | Tsunematsu *et al.,* 2000 |
|  | 27 | IRBLta-K1 | BMsf-27 | *Pita* | K1 | Tsunematsu *et al.,* 2000 |
|  | 28 | IRBLta-CT2 | BMsf-28 | *Pita* | C105TTP2L9 | Tsunematsu *et al.,* 2000 |
|  | 29 | IRBL19-A | BMsf-29 | *Pi19(t)* | Aichiasahi | Tsunematsu *et al.,* 2000 |
|  | 30 | IRBL20-IR24 | BMsf-30 | *Pi20(t)* | ARL24(IR 24) | Tsunematsu *et al.,* 2000 |
|  | 31 | IRBL11-Zh | BMsf-31 | *Pi11(t)* | Zhaiyeqing8 | Tsunematsu *et al.,* 2000 |
| LTH NILs (11 genes) | 32 | IRBLb-B[LT] | BMsf-32 | *Pib* | BL1 | Telebanco-Yanoria *et al.,* 2010 |
|  | 33 | IRBLz5-CA[LT] | BMsf-33 | *Piz-5/Pi2*(t) | C101A51 | Telebanco-Yanoria *et al.,* 2010 |
|  | 34 | IRBL9-W[LT] | BMsf-34 | *Pi9(t)* | WHD-1S-75-1-127 | Telebanco-Yanoria *et al.,* 2010 |
|  | 35 | IRBL3-CP4[LT] | BMsf-35 | *Pi3* | C104PKT | Telebanco-Yanoria *et al.,* 2010 |
|  | 36 | IRBLa-Ze[LT] | BMsf-36 | *Pia* | Zenith | Telebanco-Yanoria *et al.,* 2010 |
|  | 37 | IRBLks-S[LT] | BMsf-37 | *Pik-s* | Shin 2 | Telebanco-Yanoria *et al.,* 2010 |
|  | 38 | IRBLKs-B40[LT] | BMsf-38 | *Pik-s* | B40 | Telebanco-Yanoria *et al.,* 2010 |
|  | 39 | IRBLKs-Zh[LT] | BMsf-39 | *Pik-s* | Zhaiyeqing 8 | Telebanco-Yanoria *et al.,* 2010 |
|  | 40 | IRBLk-Ka[LT] | BMsf-40 | *Pik* | Kanto 51 | Telebanco-Yanoria *et al.,* 2010 |
|  | 41 | IRBLkh-K3[LT] | BMsf-41 | *Pik-h* | K3 | Telebanco-Yanoria *et al.,* 2010 |
|  | 42 | IRBL7-M[LT] | BMsf-42 | *Pi7(t)* | RIL29 | Telebanco-Yanoria *et al.,* 2010 |
|  | 43 | IRBLk*-Du[LT] | BMsf-43 | *Pik** | Dular | Telebanco-Yanoria *et al.,* 2010 |
|  | 44 | IRBLk*-NP[LT] | BMsf-44 | *Pik** | NP125 | Telebanco-Yanoria *et al.,* 2010 |
|  | 45 | IRBLk*-F14[LT] | BMsf-45 | *Pik** | F-14-3 | Telebanco-Yanoria *et al.,* 2010 |
|  | 46 | IRBLk*-F25[LT] | BMsf-46 | *Pik** | F25-3 | Telebanco-Yanoria *et al.,* 2010 |
|  | 47 | IRBLk*-F66[LT] | BMsf-47 | *Pik** | F-66-1 | Telebanco-Yanoria *et al.,* 2010 |
|  | 48 | IRBLta-CT2[LT] | BMsf-48 | *Pita*/*Pi4*(t) | C105TTP2L9 | Telebanco-Yanoria *et al.,* 2010 |
|  | 49 | IRBLta-K1[LT] | BMsf-49 | *Pita/Pi4(t)* | K1 | Telebanco-Yanoria *et al.,* 2010 |
|  | 50 | IRBLta-Zh[LT] | BMsf-50 | *Pita/ Pi4(t)* | Zhaiyeqing 8 | Telebanco-Yanoria *et al.,* 2010 |
|  | 51 | IRBLta2-Pi[LT] | BMsf-51 | *Pita-2* | Pi No.4 | Telebanco-Yanoria *et al.,* 2010 |
| CO39 NILs (14 genes) | 52 | CO39 | BMsf-61 | *Pia* | - | Telebanco-Yanoria *et al*., 2011 |
|  | 53 | IRBLsh-Ku[CO] | BMsf-62 | *Pish* | Kusabue | Telebanco-Yanoria *et al*., 2011 |
|  | 54 | IRBLsh-S[CO]-1 | BMsf-63 | *Pish* | Shin 2 | Telebanco-Yanoria *et al*., 2011 |
|  | 55 | IRBLsh-B[CO] | BMsf-64 | *Pish* | BL1 | Telebanco-Yanoria *et al*., 2011 |
|  | 56 | IRBLsh-Fu[CO] | BMsf-65 | *Pish* | Fukunishiki | Telebanco-Yanoria *et al*., 2011 |
|  | 57 | IRBLb-IT13[CO] | BMsf-66 | *Pib* | IRAT 13 | Telebanco-Yanoria *et al*., 2011 |
|  | 58 | IRBLz5-CA[CO] | BMsf-68 | *Piz5* | C101A51 | Telebanco-Yanoria *et al*., 2011 |
|  | 59 | IRBL5-M[CO] | BMsf-70 | *Pi5(t)* | RIL249 | Telebanco-Yanoria *et al*., 2011 |
|  | 60 | IRBLks-CO[CO] | BMsf-71 | *Pik-s* | Caloro | Telebanco-Yanoria *et al*., 2011 |
|  | 61 | IRBLk-Ku[CO] | BMsf-72 | *Pik* | Kusabue | Telebanco-Yanoria *et al*., 2011 |
|  | 62 | IRBLk-Ka[CO] | BMsf-73 | *Pik* | Kanto 51 | Telebanco-Yanoria *et al*., 2011 |
|  | 63 | IRBLkh-K3[CO] | BMsf-74 | *Pik-h* | K3 | Telebanco-Yanoria *et al*., 2011 |
|  | 64 | IRBLkm-Ts[CO] | BMsf-75 | *Pik-m* | Tsuyuake | Telebanco-Yanoria *et al*., 2011 |
|  | 65 | IRBLkp-K60[CO] | BMsf-76 | *Pik-p* | K60 | Telebanco-Yanoria *et al*., 2011 |
|  | 66 | IRBLk*-F21[CO] | BMsf-77 | *Pik** | F-21-6 | Telebanco-Yanoria *et al*., 2011 |
|  | 67 | IRBLk*-F14[CO] | BMsf-78 | *Pik** | F-14-3 | Telebanco-Yanoria *et al*., 2011 |
|  | 68 | IRBLk*-F25[CO] | BMsf-79 | *Pik** | F-25-3 | Telebanco-Yanoria *et al*., 2011 |
|  | 69 | IRBLk*-F40[CO] | BMsf-80 | *Pik** | F-40-3 | Telebanco-Yanoria *et al*., 2011 |
|  | 70 | IRBLk*-F66[CO] | BMsf-82 | *Pik** | F-66-1 | Telebanco-Yanoria *et al*., 2011 |
|  | 71 | IRBLk*-KU86[CO] | BMsf-83 | *Pik** | KU86 | Telebanco-Yanoria *et al*., 2011 |
|  | 72 | IRBLta-Ya[CO] | BMsf-86 | *Pita/ Pi4(t)* | Yashiromochi | Telebanco-Yanoria *et al*., 2011 |
|  | 73 | IRBLta-Me[CO] | BMsf-87 | *Pita/ Pi4(t)* | Metica 1 | Telebanco-Yanoria *et al*., 2011 |
|  | 74 | IRBLta2-Pi[CO] | BMsf-88 | *Pita-2* | Pi No.4 | Telebanco-Yanoria *et al*., 2011 |
|  | 75 | IRBLta2-Re[CO] | BMsf-89 | *Pita-2* | Reiho | Telebanco-Yanoria *et al*., 2011 |
|  | 76 | IRBLta2-IR64[CO] | BMsf-90 | *Pita-2* | IR 64 | Telebanco-Yanoria *et al*., 2011 |
|  | 77 | IRBL1-LA[CO] | BMsf-91 | *Pi1* | C101LAC | Telebanco-Yanoria *et al*., 2011 |
| US2 NILs (23 genes) | 78 | US-2 | BMsf-93 | Susceptible recurrent parent | - | Fukuta *et al*., 2022 |
|  | 79 | US2NILPia-Aichiasahi | BMsf-101 | *Pia* | Aichiasahi | Fukuta *et al*., 2022 |
|  | 80 | US2NILPia-C | BMsf-102 | *Pia* | CO 39 | Fukuta *et al*., 2022 |
|  | 81 | US2NILPii-F5 | BMsf-98 | *Pii* | Fujisaka 5 | Fukuta *et al*., 2022 |
|  | 82 | US2NILPi3-CP4 | BMsf-99 | *Pi3* | C104PKT | Fukuta *et al*., 2022 |
|  | 83 | US2NILPi5-M | BMsf-100 | *Pi5(t)* | RIL249 | Fukuta *et al*., 2022 |
|  | 84 | US2NILPiks-F5 | BMsf-103 | *Pik-s* | Fujisaka 5 | Fukuta *et al*., 2022 |
|  | 85 | US2NILPiks-S | BMsf-104 | *Pik-s* | Shin 2 | Fukuta *et al*., 2022 |
|  | 86 | US2NILPikm-TS | BMsf-107 | *Pik-m* | Tsuyuake | Fukuta *et al*., 2022 |
|  | 87 | US2NILPi1-CL | BMsf-106 | *Pi1* | C101LAC | Fukuta *et al*., 2022 |
|  | 88 | US2NILPikh-K3 | BMsf-105 | *Pik-h* | K3 | Fukuta *et al*., 2022 |
|  | 89 | US2NILPik-Ka | BMsf-108 | *Pik* | Kanto 51 | Fukuta *et al*., 2022 |
|  | 90 | US2NILPikp-K60 | BMsf-109 | *Pik-p* | K60 | Fukuta *et al*., 2022 |
|  | 91 | US2NILPi7(t)-M | BMsf-110 | *Pi7(t)* | RIL29 | Fukuta *et al*., 2022 |
|  | 92 | US2NILPi9-W | BMsf-94 | *Pi9* | WHD-1S-75-1-127 | Fukuta *et al*., 2022 |
|  | 93 | US2NILPiz-Fu | BMsf-95 | *Piz* | Fukunishiki | Fukuta *et al*., 2022 |
|  | 94 | US2NILPiz5-CA | BMsf-96 | *Piz-5/ Pi2(t)* | C101A51 | Fukuta *et al*., 2022 |
|  | 95 | US2NILPizt-T | BMsf-97 | *Piz-t* | Totide 1 | Fukuta *et al*., 2022 |
|  | 96 | US2NILPita2-Re | BMsf-111 | *Pita-2* | Reiho | Fukuta *et al*., 2022 |
|  | 97 | US2NILPita-K1 | BMsf-112 | *Pita/Pi4(ta)* | K1 | Fukuta *et al*., 2022 |
|  | 98 | US2NILPita-CP1 | BMsf-113 | *Pita/Pi4(ta)* | C101PKT | Fukuta *et al*., 2022 |
|  | 99 | US2NILPita-CT2 | BMsf-114 | *Pita/Pi4(ta)* | C105TTP2L9 | Fukuta *et al*., 2022 |
|  | 100 | US2NILPi20-IR24 | BMsf-115 | *Pi20(t)* | ARL24(IR 24) | Fukuta *et al*., 2022 |
|  | 101 | US2NILPi35-Hokkai188 | - | *Pi35* | Hokkai188 | Saito *et al.,* 2022 |
|  | 102 | US2NILpi21-Owarihatamochi | - | *pi21* | Owarihatamochi | Saito *et al.,* 2022 |
|  | 103 | US2NILPi37-KRIL17 | - | *Pi37-KRIL17* | LRIL17 | Saito *et al.,* 2022 |
|  | 104 | US2NILPb1-Asanohikari | - | *Pb1* | Asanohikari | Saito *et al.,* 2022 |
|  | 105 | US2NILPi34-Chubu32 | - | *Pi34 (Chubu32)* | Chubu32 | Saito *et al.,* 2022 |
|  | 106 | US2NILPi34-Chugoku40 | - | *Pi34 (Chugoku40)* | Chugoku40 | Saito *et al.,* 2022 |
|  | 107 | US2NILPiPHL9-HokkaiPL9 | - | *PiPHL9* | HokkaiPL9 | Saito *et al.,* 2022 |
| IR49830-7-1-2-2 NILs (9 genes) | 108 | IR49830-7-1-2-2 | BMsf-118 | *Pia*, *Pib*, *Pik-s*, *Pita*, and *Pi11*(t) | - | Koide *et al*. 2011 |
|  | 109 | IRBL9-W[RL] | BMsf-119 | *Pi9* | WHD-1S-75-1-127 | Koide *et al*. 2011 |
|  | 110 | IRBLz5-CA[RL] | BMsf-120 | *Piz5* | C101A51 | Koide *et al*. 2011 |
|  | 111 | IRBL3-CP4[RL] | BMsf-121 | *Pi3* | C104PKT | Koide *et al*. 2011 |
|  | 112 | IRBL5-M[RL] | BMsf-122 | *Pi5* | RIL249 | Koide *et al*. 2011 |
|  | 113 | IRBL7-M[RL] | BMsf-123 | *Pi7* | RIL29 | Koide *et al*. 2011 |
|  | 114 | IRBLk-Ku[RL] | BMsf-124 | *Pik* | Kusabue | Koide *et al*. 2011 |
|  | 115 | IRBLsh-Fu[RL] | BMsf-125 | *Pish* | Fukunishiki | Koide *et al*. 2011 |
|  | 116 | IRBLta2-Pi[RL] | BMsf-126 | *Pita2* | Pi No.4 | Koide *et al*. 2011 |
|  | 117 | IRBLsh-T[RL] | BMsf-127 | *Pish* | Totide 1 | Koide *et al*. 2011 |
| IR64 NILs (11 genes) | 118 | IR 64 | BMsf-128 | *Pia, Pik-s, Pib, Piz-t, Pi20(t) or Pita-2,Pita* + unknown) | - | Fukuta *et al.* 2022 |
|  | 119 | IR64NILPik-Ka | BMsf-129 | *Pik* | Kanto 51 | Fukuta *et al.* 2022 |
|  | 120 | IR64NILPik-Ka(ML) | BMsf-130 | *Pik* | IRBLk-Ka | Fukuta *et al.* 2022 |
|  | 121 | IR64NILPik-Ka(CO) | BMsf-131 | *Pik* | IRBLk-Ka[CO] | Fukuta *et al.* 2022 |
|  | 122 | IR64NILPikh-K3 | BMsf-132 | *Pikh* | K3 | Fukuta *et al.* 2022 |
|  | 123 | IR64NILPikh-K3(CO) | BMsf-133 | *Pikh* | IRBLkh-K3[CO] | Fukuta *et al.* 2022 |
|  | 124 | IR64NILPikp-K60 | BMsf-134 | *Pikp* | K60 | Fukuta *et al.* 2022 |
|  | 125 | IR64NILPikp-K60(CO) | BMsf-135 | *Pikp* | IRBLkp-K60[CO] | Fukuta *et al.* 2022 |
|  | 126 | IR64NILPi1-CL(ML) | BMsf-136 | *Pi1* | IRBL1-CL | Fukuta *et al.* 2022 |
|  | 127 | IR64NILPi1-CL(CO) | BMsf-137 | *Pi1* | IRBL1-LA[CO] | Fukuta *et al.* 2022 |
|  | 128 | IR64NILPi7-M(ML) | BMsf-138 | *Pi7(t)* | IRBL7-M | Fukuta *et al.* 2022 |
|  | 129 | IR64NILPi7-M(CO) | BMsf-139 | *Pi7(t)* | IRBL7-M[CO] | Fukuta *et al.* 2022 |
|  | 130 | IR64NILPiz-Fu(ML) | BMsf-140 | *Piz* | IRBLz-F | Fukuta *et al.* 2022 |
|  | 131 | IR64NILPiz5-CA(ML) | BMsf-141 | *Piz-5* | IRBLz5-CA | Fukuta *et al.* 2022 |
|  | 132 | IR64NILPiz5-CA(CO) | BMsf-142 | *Piz-5* | IRBLz5[CO] | Fukuta *et al.* 2022 |
|  | 133 | IR64NILPi9-W | BMsf-143 | *Pi9* | WHD-1S-75-1-127 | Fukuta *et al.* 2022 |
|  | 134 | IR64NILPi9-W(ML) | BMsf-144 | *Pi9* | IRBL9-W | Fukuta *et al.* 2022 |
|  | 135 | IR64NILPish-S(CO) | BMsf-146 | *Pish* | IRBLsh-S[CO] | Fukuta *et al.* 2022 |
|  | 136 | IR64NILPish-Ku(CO) | BMsf-147 | *Pish* | IRBLsh-Ku[CO] | Fukuta *et al.* 2022 |
|  | 137 | IR64NILPish-S(ML) | BMsf-148 | *Pish* | IRBLsh-Ku | Fukuta *et al.* 2022 |
|  | 138 | IR64NILPii-F5(ML) | BMsf-149 | *Pii* | IRBLi-F5 | Fukuta *et al.* 2022 |
|  | 139 | IR64NILPi3-CP4(ML) | BMsf-150 | *Pi3* | IRBL3-CP4 | Fukuta *et al.* 2022 |
| *: an unknown Pik allele as indicated by the original publication; -: dont have | | | | | | |
